# Predicting Optimal Outcomes in Cognitive Therapy or Interpersonal Psychotherapy for Depressed Individuals Using the Personalized Advantage Index Approach

**DOI:** 10.1371/journal.pone.0140771

**Published:** 2015-11-10

**Authors:** Marcus J. H. Huibers, Zachary D. Cohen, Lotte H. J. M. Lemmens, Arnoud Arntz, Frenk P. M. L. Peeters, Pim Cuijpers, Robert J. DeRubeis

**Affiliations:** 1 Department of Clinical Psychology, VU University Amsterdam, Amsterdam, The Netherlands; 2 Department of Psychology, University of Pennsylvania, Philadelphia, United States of America; 3 Department of Clinical Psychological Science, Maastricht University, Maastricht,The Netherlands; 4 Department of Clinical Psychology, University of Amsterdam, Amsterdam, The Netherlands; 5 Department of Psychiatry, Maastricht University, Maastricht, The Netherlands; Istituto Superiore di Sanità, ITALY

## Abstract

**Introduction:**

Although psychotherapies for depression produce equivalent outcomes, individual patients respond differently to different therapies. Predictors of outcome have been identified in the context of randomized trials, but this information has not been used to predict which treatment works best for the depressed individual. In this paper, we aim to replicate a recently developed treatment selection method, using data from an RCT comparing the effects of cognitive therapy (CT) and interpersonal psychotherapy (IPT).

**Methods:**

134 depressed patients completed the pre- and post-treatment BDI-II assessment. First, we identified baseline predictors and moderators. Second, individual treatment recommendations were generated by combining the identified predictors and moderators in an algorithm that produces the Personalized Advantage Index (PAI), a measure of the predicted advantage in one therapy compared to the other, using standard regression analyses and the leave-one-out cross-validation approach.

**Results:**

We found five predictors (gender, employment status, anxiety, personality disorder and quality of life) and six moderators (somatic complaints, cognitive problems, paranoid symptoms, interpersonal self-sacrificing, attributional style and number of life events) of treatment outcome. The mean average PAI value was 8.9 BDI points, and 63% of the sample was predicted to have a clinically meaningful advantage in one of the therapies. Those who were randomized to their predicted optimal treatment (either CT or IPT) had an observed mean end-BDI of 11.8, while those who received their predicted non-optimal treatment had an end-BDI of 17.8 (effect size for the difference = 0.51).

**Discussion:**

Depressed patients who were randomized to their predicted optimal treatment fared much better than those randomized to their predicted non-optimal treatment. The PAI provides a great opportunity for formal decision-making to improve individual patient outcomes in depression. Although the utility of the PAI approach will need to be evaluated in prospective research, this study promotes the development of a treatment selection approach that can be used in regular mental health care, advancing the goals of personalized medicine.

## Introduction

Of all the effective psychotherapies for depression, cognitive therapy (CT) and interpersonal psychotherapy (IPT) are most prominent and extensively researched. A recent meta-analysis [1] identified five head-to-head comparisons of CT and IPT. In three studies [2–4], individual therapy was studied and in the remaining two a group format was used [5, 6]. Results showed no significant differences between CT and IPT, although severely depressed patients fared better in IPT in one study [2] and better in CT in another [3].

Despite the fact that these psychotherapies produced equivalent outcomes on average, individual patients might respond differently to different therapies. Prediction studies have indeed identified differential treatment effects for subgroups of patients. Before we review some of those findings in the context of this paper, it is important to highlight the distinction between *prognostic factors* (predictors) and *prescriptive factors* (moderators). Prognostic variables derive from designs that hold treatment constant (or ignore differences in modality) and seek to determine whether individual differences measured at baseline predict subsequent variation in response [7]. Prognostic indices tell us which patients benefit most from a given treatment or set of treatments, but not which of two or more treatments is likely to be the best one for a given patient. Prescriptive variables on the other hand derive from comparative treatment designs and seek to determine whether individual baseline differences predict subsequent variation in response as a function of treatment type [7]. Prescriptive indices can in principle be used to guide treatment decisions for a given patient.

The National Institute of Mental Health Treatment of Depression Collaborative Research Project (TDCRP) compared the effects of CT, IPT, antidepressants (ADM) with clinical management, and placebo with clinical management and found no overall outcome differences among the groups [2], although a secondary analysis of the data indicated that CT was less efficacious than ADM and IPT in the more severely depressed patients in their sample [2, 8]. Sotsky et al. investigated other potential predictors and moderators of outcome in a multivariate analysis [9]. They found six predictors: social dysfunction; cognitive dysfunction; (low) expectation of improvement; “endogenous” depression; double depression; and duration of current episode. They also identified three moderators of treatment outcome relative to pill-placebo; patients with low social dysfunction showed a better (specific) response to IPT; patients with low cognitive dysfunction showed a better (specific) response to CT or ADM; and patients with high work dysfunction showed a better (specific) response to ADM. None of these factors predicted differential response among the active treatments.

Luty et al. compared the effectiveness of CT and IPT for major depression and found no differences in outcome between the two psychotherapies in the full sample, although severely depressed patients responded better to CT than to IPT [3], suggesting severity is a moderator of response. A subsequent multivariate prediction analysis identified three other predictors and one moderator [10]: a single episode of depression (versus recurrent depression), a higher perceived logicalness of therapy, and a moderate belief that childhood factors caused the depression were all associated with better overall outcomes post-treatment (predictors), whereas patients with more comorbid personality disorder symptoms did better in CT than in IPT (moderators) [11].

Using prescriptive indices to predict and select the best available treatment for a given individual (what works best for whom?) has been referred to as *personalized medicine*, and is considered to be one of the major challenges in health care research today [12]. Personalized medicine requires that the individual differences that predict different outcomes in different treatments are known. Simon and Perlis reviewed the available evidence relevant to personalized medicine for depression and found that only a few studies examined true moderators of outcome [13], in part because the statistical power of most existing trials is too limited to generate the interactions that reveal moderation. Some clinical characteristics, such as the presence of a personality disorder, might inform the choice between antidepressants and psychotherapy, or the choice between CT and IPT. Others have proposed that the preferences of the individual should guide treatment selection, especially in the choice between antidepressants and psychotherapy, but the results from studies assessing the impact of patient preference on treatment outcomes in depression have been mixed, mostly showing no association between preference and outcome [14]. Given the large number of trials comparing different antidepressant medications with each other, surprisingly little data guide treatment selection of specific antidepressants. Cuijpers et al. concluded in their review of the literature that the development of personalized medicine in this area has just begun [15].

But even if more moderators are identified in future studies, how can we actually use this information to prescribe the best available treatment to individual patients? Most prediction studies stop at reporting the predictors or moderators considered in isolation. When multivariate analyses are presented, it is often to show that the individual variables continue to exhibit an interaction effect with treatment condition, even when other variables are in the model. Clinicians are then left with no guidance as to how to use this information, especially if multiple moderators point in different directions [16]. An exception to the rule is a prediction analysis by Barber and Muenz based on the TDCRP data [17]. In this paper, patients are matched to treatment (CT versus IPT) using a matching factor that combined three prescriptive indices (i.e. marital status, avoidant personality style, obsessive personality style) in a regression formula, indicating a preference for CT (positive matching factor) or IPT (negative matching factor) for individual patients.

Nearly 20 years later, DeRubeis et al. [16] returned to the subject of treatment selection. Noting that the statistical approach and clinical recommendations made by Barber and Muenz (1996) had not been followed up in subsequent years, they introduced a new method to combine multiple predictors and moderators in a statistical model that produces the Personalized Advantage Index (PAI). This PAI identifies the treatment that is predicted to be more efficacious for an individual patient, and it provides an index that reflects the magnitude of the predicted advantage. Using pre-identified predictors and moderators [18–20] from a large-scale trial comparing antidepressant medication (ADM) and cognitive therapy for depression [21], it was found that for 60% of the sample a “clinically significant advantage” was predicted in either ADM or CT, compared to the other treatment. Moreover, among those patients with a clinically significant advantage predicted, those who had been randomized to their predicted optimal treatment evidenced far better outcomes than those randomized to their predicted non-optimal treatment, with a significant effect size of 0.58 between the groups.

In this paper, we aim to replicate the treatment selection method that DeRubeis et al. [16] introduced, but this time by comparing two theoretically distinct psychotherapies that are known to result in overall equivalent outcomes [1]. Data come from a recently published trial in which we found no significant differences between CT and IPT for depression in the acute phase [22]. First, we identified predictors and moderators using a modified domain approach [19] that was replicated successfully [23]. Second, we combined these prescriptive indices in models that we used to predict optimal treatment outcomes in individual trial patients, employing the PAI approach as a post-hoc analysis.

## Methods

### Design and participants

Data come from a single-center RCT into the effectiveness and mechanisms of change of individual CT and IPT for depression [22]. Details about the study design have been fully described elsewhere [24]. A total of 182 depressed outpatients were randomly assigned to (a) CT (*n* = 76), (b) IPT (*n* = 75), (c) or a 2-month waiting-list control condition followed by treatment of choice (*n* = 31). Only the two active groups are part of the present analysis, as are the data collected pre-treatment at baseline and post-treatment (after 7 months). The primary outcome measure was depression severity as measured with the Beck Depression Inventory-II [25]. The Medical Ethics Committee of Maastricht University approved the study protocol, and all participants provided written informed consent. The study is registered at the Netherlands Trial Register, part of the Dutch Cochrane Centre (ISRCTN67561918).

Participants were adult outpatients (18–65 years) referred to the mood disorder program of the Academic Community Mental Health Centre Maastricht with a primary diagnosis of MDD, confirmed by the Structured Clinical Interview for DSM-IV Axis I disorders (SCID-I) [26]. Further inclusion criteria were: internet access, an e-mail address, and sufficient knowledge of the Dutch language. Exclusion criteria were: bipolar or chronic (current episode > 5 years) depression, elevated acute suicide risk, current use of antidepressant medication, concomitant psychological treatment, drug and/or alcohol abuse or dependence, and mental retardation (IQ < 80).

### Treatment

Treatment (CT or IPT) contained 12 to 20 individual sessions of 45 minutes, depending on the individual progress of patients. CT was based on the manual by Beck et al. [27], and IPT followed the guidelines by Klerman et al. [28].

The ten therapists (five in each condition) that delivered the interventions were licensed psychologists, psychotherapists and psychiatrists who received adequate training in CT and/or IPT, with an average of 9.1 years of clinical experience (range 4 to 21 years). To prevent contamination, therapists were uniquely assigned to either CT or IPT. Quality of therapy was rated by independent assessors as being good to excellent in both conditions [22].

### Measures

The Beck Depression Inventory II (BDI-II) [25] assesses the severity of depression symptoms. In this analysis, baseline (0 months) and post-treatment (7 months) scores on the BDI-II were used. Treatment condition was a binary variable and referred to either cognitive therapy (CT) or interpersonal therapy (IPT).

Putative predictors were all measured at baseline and are summarized in Table 1. Instruments used to assess these predictors included the Attributional Style Questionnaire (ASQ); the Beck Hopelessness Scale (BHS); the Brief Symptom Inventory (BSI); the Dysfunctional Attitudes Scale-17 (DAS17); the EuroQol; the Inventory of Interpersonal Problems (IIP); the Leiden Index of Cognitive Reactivity (LEIDS); the Quick Inventory of Depressive Symptoms (QIDS); the RAND-36; the Ruminative Response Scale (RRS); the Structured Clinical Interview for DSM-IV axis I Disorders (SCID-I); the Structured Clinical Interview for DSM-IV axis II Disorders (SCID-II); the Self-Liking and Self-Competence Scale (SLSC-R); and the Work and Social Adjustment Scale (WSAS) (for a complete description and references to these instruments, we refer to our design paper [24]).

**Table 1:** Variables per domain.

**Domain 1: Depression**
Previous episodes (0 = no, 1 = yes)
Depression severity (QIDS)
Hopelessness–Feelings about the future (BHS)
Hopelessness–Loss of motivation (BHS)
Hopelessness–Future expectations (BHS)
**Domain 2: Demographics**
Gender (0 = male, 1 = female)
Age
Marital status (1 = no partner, 0 = partner)
Employment status (1 = no active employment, 0 = active employment)
Treatment expectancy (0 = not successful– 10 = very successful)
**Domain 3: Psychological Distress**
General Psychological Distress–Somatic Complaints (BSI)
General Psychological Distress–Cognitive Problems (BSI)
General Psychological Distress–Interpersonal Sensitivity (BSI)
General Psychological Distress–Depression (BSI)
General Psychological Distress–Anxiety (BSI)
General Psychological Distress–Hostility (BSI)
General Psychological Distress–Phobic Anxiety (BSI)
General Psychological Distress–Paranoid Symptoms (BSI)
General Psychological Distress–Psychoticism (BSI)
Number of Comorbid Axis I disorder(s) (SCID-I)
Personality disorder (SCID-II; 0 = no, 1 = yes)
Personality disorder traits (SCID-II; 0 = no, 1 = yes)
**Domain 4: General Functioning**
Social and work functioning (WSAS)
Level of Impairment–Physical functioning (RAND-36)
Level of Impairment–Social functioning (RAND-36)
Level of Impairment–Role limitations (physical problems) (RAND-36)
Level of Impairment–Role Limitations (emotional problems) (RAND-36)
Level of Impairment–Mental health (RAND-36)
Level of Impairment–Vitality (RAND-36)
Level of Impairment–Pain (RAND-36)
Level of Impairment–General health perception (RAND-36)
Level of Impairment–Perceived health change during past year (RAND-36)
Quality of life Utility Score (EuroQol)
**Domain 5: Psychological Processes**
Dysfunctional Attitudes–Factor 1 (DAS17)
Dysfunctional Attitudes–Factor 2 (DAS17)
Interpersonal Problems–Domineering (IIP)
Interpersonal Problems–Vindictive (IIP)
Interpersonal Problems–Cold/Distant (IIP)
Interpersonal Problems–Socially Inhibited (IIP)
Interpersonal Problems–Nonassertive (IIP)
Interpersonal Problems–Overly accommodating (IIP)
Interpersonal Problems–Self-sacrificing (IIP)
Interpersonal Problems–Intrusive/Needy (IIP)
Self-esteem–Self Liking (SLSC-R)
Self-esteem–Self Competence (SLSC-R
Cognitive Reactivity–Hopelessness (LEIDS)
Cognitive Reactivity–Acceptance (LEIDS)
Cognitive Reactivity–Aggression (LEIDS)
Cognitive Reactivity–Control (LEIDS)
Cognitive Reactivity–Risk aversion (LEIDS)
Cognitive Reactivity–Rumination (LEIDS)
Rumination (RRS)
Attributional Style–Achievement (ASQ)
Attributional Style–Affiliation (ASQ)
**Domain 6: Life and Family History**
Number of life events in life
Number of life events in past year
Parental Illness–One or both parents in treatment (0 = yes, 1 = no)
Parental Depression–One or both parents with depression (0 = yes, 1 = no)
Parental Anxiety–One or both parents with anxiety disorder (0 = yes, 1 = no)
Parental Alcohol Abuse–One or both parents with alcohol abuse, (0 = yes, 1 = no)
Parental Suicidality–One or both parents with suicidality (0 = yes, 1 = no)

ASQ = Attributional Style Questionnaire; BHS = Beck Hopelessness Scale; BSI = Brief Symptom Inventory; DAS17 = Dysfunctional Attitudes Scale 17; EuroQol = Quality of Life—based on societal appreciation of health condition; IIP = Inventory of Interpersonal Problems; LEIDS = Leiden Index of Cognitive Reactivity; QIDS = Quick Inventory of Depressive Symptoms; RAND-36 = Quality of Life—impairments due to physical and mental health status; RRS = Ruminative Response Scale; SCID-I = Structured Clinical Interview for DSM-IV axis I Disorders; SCID-II = Structured Clinical Interview for DSM-IV axis II Disorders; SLSC-R = Self-Liking and Self-Competence Scale; WSAS = Work and Social Adjustment Scale.

### Variable transformation

For 134 participants (89% of total sample) BDI-II post-treatment scores (7 months) were available. We used the square root of the BDI in all analyses because tests showed the residuals of the raw BDI scores were non-normally distributed, whereas the transformed variable yielded model residuals that were not significantly different from a normal distribution. Following the recommendations of Kraemer et al. [29], we centered the treatment variable at -0.5 (CT) and +0.5 (IPT). All predictor variables that were binary were also centered -0.5 and + 0.5, and continuous variables were mean-centered.

### Variable selection

To identify predictors and moderators in this sample, we adopted the principles of the domain approach outlined by Fournier et al. [19]. First, we grouped potential predictors in separate domains (Table 1). In the depression domain, for example, we grouped predictors that relate to the depression concept. We built separate linear regression models for each domain, and each model included all the domain variables and their interaction with treatment (CT or IPT), as well as the main effect of treatment and BDI baseline.

In step 1, for each domain model, the variables that were significant at a threshold of *α* = 0.2 were carried into step 2. The main effect of treatment and baseline BDI were always included in every model (despite the fact that treatment main effect was non-significant). If an interaction between a variable and treatment was significant, the main effect was carried through to the next step regardless of whether or not it was significant. In step 2, a new model was built with main effects of treatment and baseline BDI, as well as any main effects and interactions that were carried through from step 1. The same significance threshold process was then applied using the new threshold value of *α* = 0.1. In Step 3, the same process was repeated but with the threshold of *α* = 0.05. Any interactions that were significant at *α* = 0.05 were carried into the final model with their main effects (regardless of significance), as were any main effects that were significant. The final model was then created using the main effect of treatment and baseline BDI, as well as any interactions or main effects that emerged from step 3 of the domain approach–combining all of the variables from step 3 from all of the domains.

### Building the Personalized Advantage Index

The leave-one-out cross-validation (LOO) approach [30, 31] was used so that a new model, containing all previously identified predictors and moderators, was built for each individual patient. The regression formula then looks like this:*SQRT* (End-BDI) = k + BDI pre + Tx (CT or IPT) + prognostic (main) + prescriptive (Tx * main). This creates an analogous situation to what would happen if we built a model on a group of patients in a clinic and then a new patient walked in the door. It protects against overfitting by ensuring that a patient’s data cannot contribute to or inform the model that will be used to predict that patient’s outcome. A more detailed description of the approach can be found elsewhere [16]. A new model (using the exact same set of variables) is built for the 134^th^ patient using data from the other 133 patients. Next, the 134^th^ patient’s observed values on all the variables in the model are entered into each of two models, one in which treatment is set to -0.5 (CT) and one in which treatment is set to 0.5 (IPT). The prediction generated for the treatment the patient actually received, the factual prediction, is produced by inputting all the real values for that patient, including their treatment assignment. The counterfactual prediction is produced by substituting the opposite value of the treatment variable (e.g., from -0.5 to +0.5) wherever it appears in the model (i.e., as main effect and in all treatment by predictor interaction terms). The factual and counterfactual predictions are then squared to convert them back to units of the BDI. The predicted end of treatment BDI score in CT minus the predicted end of treatment BDI score in IPT is termed the PAI, and represents an index of the expected advantage of one treatment over another. The sign of the PAI indicates which treatment is predicted to be optimal for a patient. A negative PAI indicates that the first treatment (CT) is predicted to be optimal, whereas a positive PAI indicates that the second treatment (IPT) is predicted to be optimal. Higher absolute values of PAIs indicate a stronger predicted benefit of one treatment over the other.

Note that for any given individual, both prognostic variables (predictors) and prescriptive variables (moderators) are used to derive the PAI for reasons of precision, and add to the predicted end-score on the BDI. However, only the moderators in the equation are responsible for the magnitude of an individual’s PAI, as these factors are indicators of the predicted difference in CT and IPT.

## Results

### Effectiveness of cognitive therapy and interpersonal psychotherapy

Effectiveness findings were published in a previous paper [22] and showed that both CT (*n* = 76) and IPT (*n* = 75) were effective interventions, compared to waiting-list control condition (*n* = 31). Depressive symptoms decreased significantly in the 7-month course of both therapies (Cohen’s *d* for within-group effect size of 1.71 for CT and 1.72 for IPT), and the symptom reduction remained stable in the 5 months following treatment termination. No significant differences between CT and IPT were observed.

### Identification of predictors and final prediction model

Of the 151 patients who were randomized to CT or IPT, 134 patients (CT = 69; IPT = 65) provided post-treatment BDI scores (end-BDI) and were included in the present analysis. In total 61 potential predictors at baseline and their interaction with treatment condition (CT or IPT) were evaluated within 6 domains (see Table 1 for an overview). Table 2 presents the variables that were used in the prediction models. Five predictors (main effects) and six moderators (interaction effects) emerged as significant at *α* = 0.05 within their separate domains, and were thus included in the final prediction model. Note that some of these predictors and moderators did not remain significant when entered in the final model.

**Table 2:** Final Prediction Model.

Predictors	ß	t-statistic	p-value
*Treatment*			
BDI baseline	0,07	3,10	0,00
Treatment (CT or IPT)	-0,03	-0,11	0,92
*Predictors*			
Gender	0,31	1,04	0,30
Employment Status	-0,53	-2,02	0,05
BSI Somatic Complaints	-0,01	-0,31	0,75
BSI Cognitive Problems	-0,01	-0,36	0,72
BSI Anxiety	0,08	2,16	0,03
BSI Paranoid Symptoms	0,01	0,36	0,72
Personality Disorder (SCID-II)	-0,52	-1,93	0,06
Quality of life Utility Score	-1,10	-1,93	0,06
IIP Self-sacrificing	0,01	0,20	0,84
ASQ Achievement	-0,08	-0,73	0,47
Number of life events in past year	-0,08	-0,85	0,40
*Moderators*			
BSI Somatic Complaints x Treatment	0,08	1,27	0,21
BSI Cognitive Problems x Treatment	-0,28	-3,86	0,00
BSI Paranoid Symptoms x Treatment	0,17	2,20	0,03
IIP Self-sacrificing x Treatment	0,10	1,94	0,05
ASQ Achievement x Treatment	0,40	1,88	0,06
Number of life events in past year x Treatment	0,43	2,21	0,03

Female gender, being actively employed, low anxiety scores, the absence of a personality disorder and a high quality of life all predicted lower depression symptoms after treatment, irrespective of the therapy received. These were the prognostic variables, represented in the models as main effects only.

Somatic complaints, cognitive problems, paranoid symptoms, interpersonal self-sacrificing (i.e., caring for others, even when it requires sacrificing one’s own needs), attributional style focused on achievement goals and the number of life events in the past year predicted a differential response in CT and IPT, with cognitive problems predicting a better response to IPT and the other five moderators predicting a better response to CT.

### Predicted outcomes and PAIs

**Fig 1:**
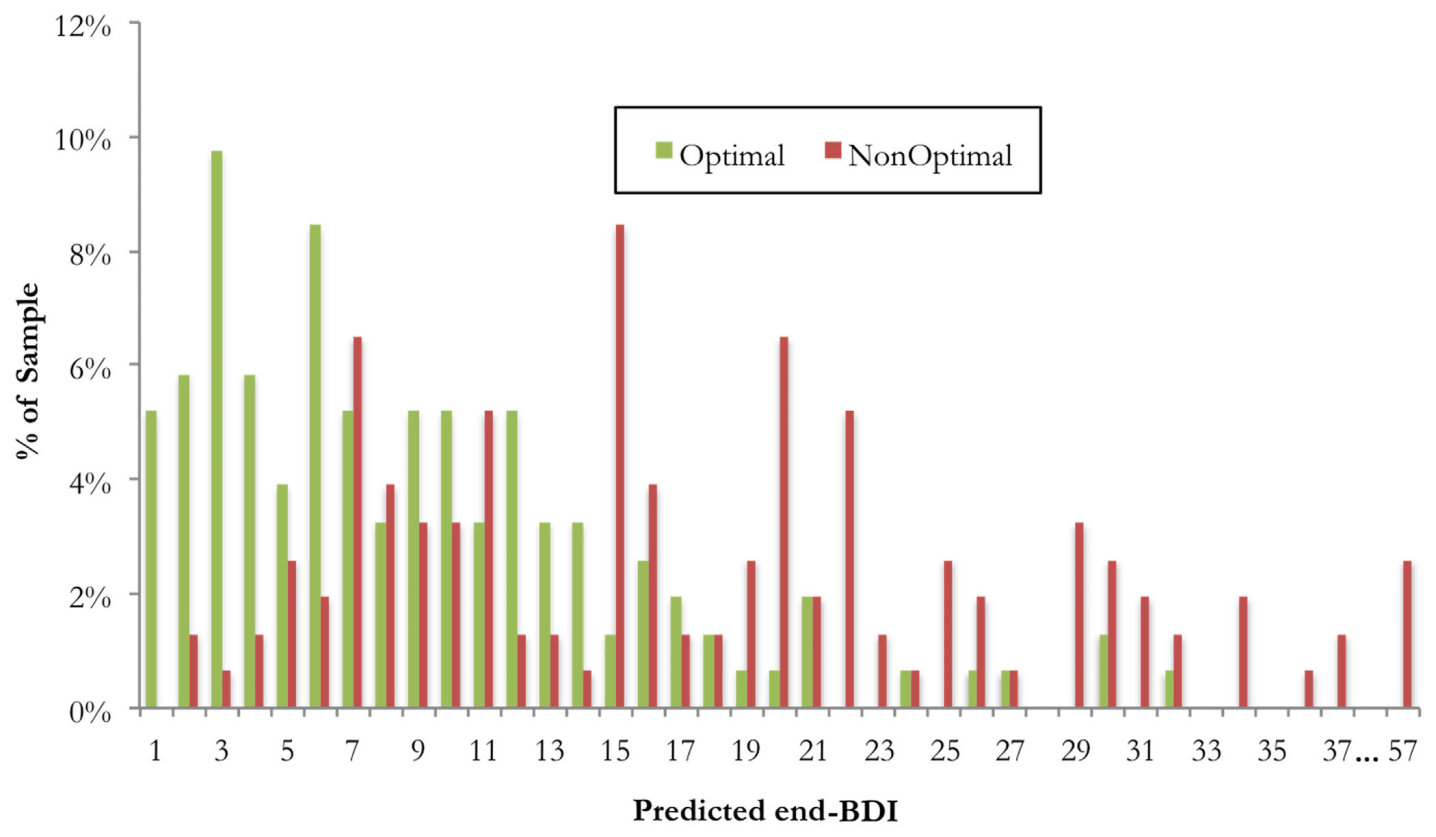
Frequency histogram showing predicted end-BDI scores for each patient in their Optimal and their Non-Optimal treatment, as indicated by the treatment selection algorithm.

Using the identified predictors and moderators, Personalized Advantage Indexes (PAI) were calculated for each individual patient, by subtracting the predicted outcomes in each of the two therapies for that patient. We refer to the therapy that is predicted to produce the greater benefit for the patient as the optimal treatment, whereas the other therapy is referred to as non-optimal treatment. In Fig 1, we present the frequency of predicted end-BDI scores in both the optimal and non-optimal treatment for every individual patient, a lower BDI score representing a more favorable outcome.

**Fig 2:**
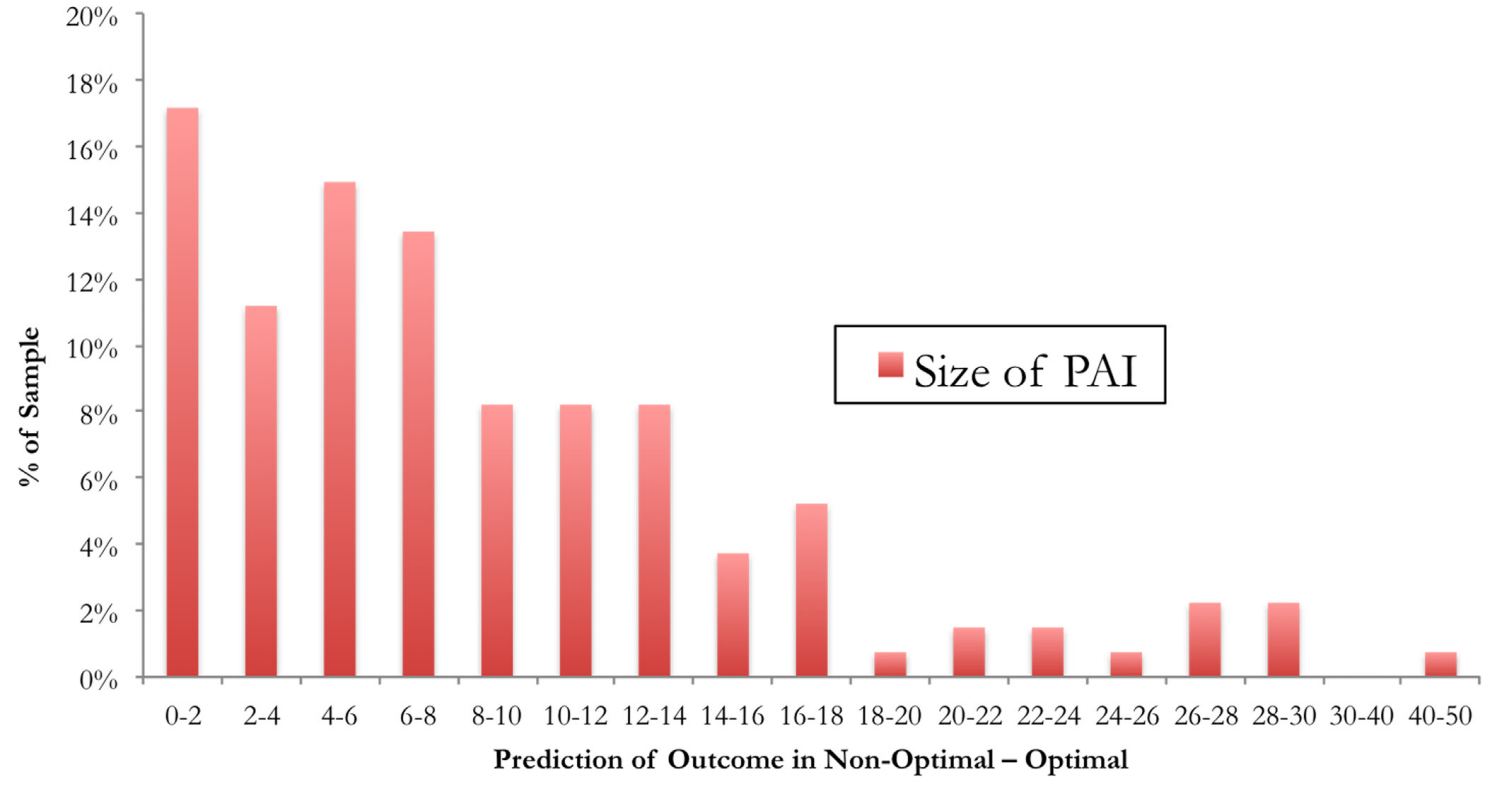
Frequency histogram showing Personalized Advantage Index (PAI) scores for all patients in the sample.

In Fig 2, the actual sizes of the PAIs we calculated are presented according to frequency. The average absolute value of the PAIs was 8.9 (7.7), which means an average 8.9 point difference predicted on the BDI between optimal (predicted mean = 8.5; sd = 6.6) and non-optimal treatment (predicted mean = 17.3; sd = 10.1). Approximately 17% of the PAIs fell between 0 and 2, indicating a small or negligible predicted advantage of one therapy over the other. However, the PAI was 5 or greater for 63% of the patients in the sample, indicating that a substantial difference was predicted between the two treatments on the BDI [32].

### Observed outcomes in optimal and non-optimal treatment

**Fig 3:**
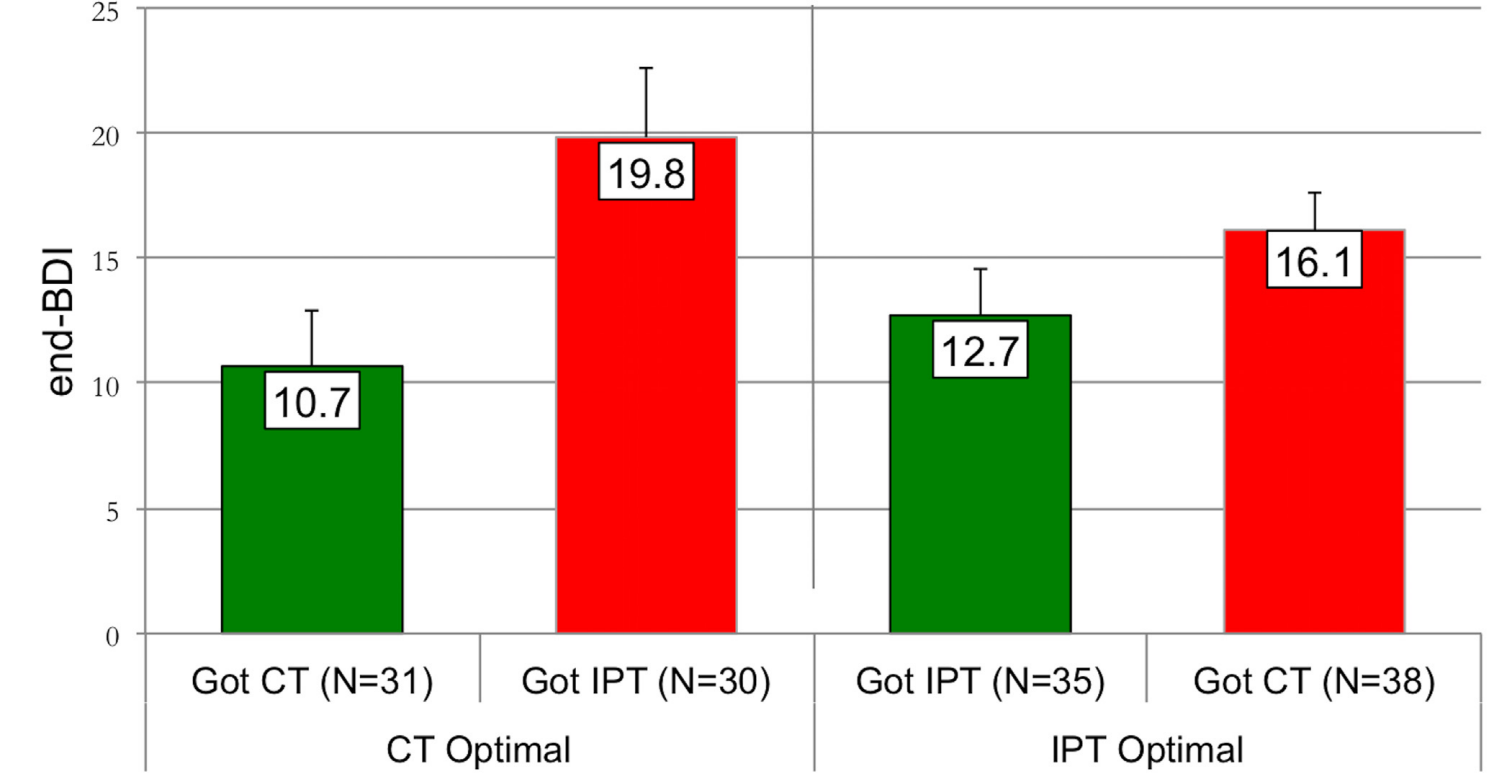
Comparison of observed mean end-BDI scores for patients randomly assigned to their Optimal treatment versus those assigned to their Non-Optimal treatment, by psychotherapy type.

Fig 3 presents the observed outcomes of the patients in our sample, stratified according to their predicted optimal treatment and the actual treatment they were randomized to. For those who were predicted to do better in CT (CT-Optimal) and who actually received CT, the observed mean end-BDI was 10.7 (sd = 12.3; *n* = 31), whereas for those in the CT-Optimal group who received IPT, the mean end-BDI was 19.8 (sd = 15.2; *n* = 30). The effect size estimate (Cohen’s *d*) for this difference is 0.66. A similar, albeit less strong difference was observed in the IPT-Optimal patients. The mean end-BDI for those who received IPT was 12.7 (sd = 10.8; *n* = 35), whereas IPT-Optimal patients who received CT had a mean end-BDI of 16.1 (sd = 8.9; *n* = 38, effect size for the difference is 0.34).

On an aggregated level, those who received their predicted optimal treatment (either CT or IPT) had an observed mean end-BDI of 11.8 (sd = 11.7; *n* = 66), whereas those who received their predicted non-optimal treatment had an observed mean end-BDI of 17.8 (sd = 12.1; *n* = 68). The effect size for this difference is 0.51.

## Discussion

### Main findings

In this study, we aimed to identify predictors and moderators of outcome in two evidence-based psychotherapies for depression, cognitive therapy and interpersonal therapy, that yielded similar overall distributions of outcomes in a randomized comparative trial. We further aimed to use these prognostic and prescriptive indices to identify the predicted optimal treatment option for each individual patient, to provide an estimate of the advantage that might be gained by applying a multivariate-based treatment selection procedure prospectively.

Using a state-of-the-art data analytic approach, we were able to identify five predictors and six moderators of outcome. Female gender, active employment, low anxiety, the absence of a personality disorder and high quality of life were all indicators of a favorable prognosis. Somatic complaints, cognitive problems, paranoid symptoms, interpersonal self-sacrificing, attributional style (achievement) and the number of life events in the past year were indicators of a differential treatment response; cognitive problems predicted a better response to IPT, while the other five moderators predicting a better response to CT. When compared to previously reported predictors and moderators, we find little overlap. Sotsky et al. [9] reported cognitive dysfunction as both a predictor and a moderator, but only between the active treatments CT and ADM in comparison to pill-placebo. Carter et al. [10] found that patients with more comorbid personality disorder symptoms responded better to CT than to IPT, while we identified the absence of personality disorder only as a general prognostic factor.

We then replicated the procedure as described by DeRubeis et al. [16] to build a treatment selection algorithm using the identified predictors and moderators, and calculate the PAIs for all individual patients in our sample. PAI sizes had a considerable range, with 63% of our sample having a PAI larger than 5, in units of the BDI, which corresponds to a clinically meaningful advantage. The distribution of observed outcomes for those who had been randomized to the psychotherapy they were predicted to benefit the most from (optimal treatment) was substantially better than for those assigned to their non-optimal treatment. The effect size estimate for the difference was 0.51.

### Interpretation

This is the first successful replication of the PAI approach that was developed by DeRubeis et al. [16]. Within the context of a randomized trial comparing CT and antidepressant medication (ADM), they combined four predictors (depression severity; chronicity; age; IQ) and five moderators (marital status; employment status; comorbid personality disorder; number of life stresses; number of prior ADM trials) of outcome into a treatment selection algorithm, and found that those assigned to their predicted optimal treatment fared much better than those assigned to their non-optimal treatment.

The effect size estimate they presented for the difference in observed outcome between the optimal and non-optimal groups in the subgroup of patients who showed a clinically meaningful advantage was 0.58, similar to the effect size we estimated in the entire sample in the present study. Cognitive therapy was one of the treatments in each of these studies, but the treatment to which it was compared was very different in the two studies: antidepressant medications in the DeRubeis study and interpersonal therapy in the present case. Thus, it was not expected that the same variables would feature in the respective prescriptive models. The magnitude of the effect sizes in both studies does suggest that combining a relatively large number of moderators into a treatment selection algorithm leads to substantial differences in predicted outcomes, on average and for a majority of the individual patients. Moreover, the differences in observed outcomes we found between the optimal and the non-optimal group are large and clinically relevant, especially considering that most available depression treatments, both psychological and pharmacological, yield similar outcomes [33], as did the treatments in the present study, in aggregate.

Finding moderators of the effect of two equally effective psychotherapies might point to different mechanisms of change [34], in line with the theorized therapy models or with other causal pathways. The moderators that we identified cannot be directly translated or interpreted as fitting the mechanisms of one therapy model or the other. However, whatever the active mechanisms in the two psychotherapies are, they seem to yield different outcomes for different subgroups of patients.

Noteworthy in this respect is that the effect size for the observed outcomes in those who received their predicted optimal treatment versus those who received their predicted non-optimal treatment was twice as high in CT (0.66) compared to IPT (0.34). This suggest that for those who are predicted to do well in CT, the expected impact of receiving CT is bigger on average than the impact of receiving IPT for those who are predicted to do well in IPT. As the moderators that add to the calculation of the PAI might point to underlying mechanisms of change, the difference in effect size between CT and IPT might indicate a difference in the importance and magnitude of certain change processes, or the therapeutic procedures that bring about change. Future research on the underlying mechanisms of change and the associated change procedures in CT and IPT could shed more light on this.

### Methodological considerations

We were able to replicate the PAI treatment selection approach using data from an RCT, while protecting against the risk of statistical overfitting, which is one of the major advantages of the leave-one-out method (LOO) we applied. In a similar vein however, one could argue that the LOO approach should also be used to identify predictors and moderators, especially if these are used to generate individual treatment recommendations in a subsequent step. Future research should explore the use of LOO predictor selection, and weigh the advantages and disadvantages of this method against those of less complicated approaches like the one presented here. It should also be noted that we replicated the PAI method, and not the findings that were reported by DeRubeis et al [16] specifically. The generalization of the predictors and moderators we found is therefore limited, as they have yet to confirmed in a subsequent study comparing CT and IPT.

Despite the advantages of LOO, the use of data from an existing RCT to generate treatment recommendations has its limitations, most importantly combining predictions and actual observations within the same study population and time frame. Our analysis demonstrating how PAI-derived predictions are associated with observed end-scores for the participants in our trial is a post-hoc analysis, much like unplanned subgroup analyses in the context of an RCT. The ultimate validation of the PAI approach will come from prospective tests in which predictors and moderators are identified in one cohort of patients, after which the treatment selection algorithm is tested in a new cohort of patients seeking health care, for example by randomizing them to their predicted optimal treatment versus “allocation as usual” or a treatment the patient prefers. For this purpose, prospective testing within routine practice settings appears most suitable and informative. Predictors and moderators of treatment outcome reported in the literature so far showed only limited overlap. Many factors could account for this heterogeneity, including differences in study populations, study designs, measurement instruments and data analytic approaches. Predictive indices might therefore vary between populations, stressing the need for population-specific treatment selection algorithms. We believe that, ultimately, patient data routinely assessed in a given mental health clinic can be used to build and implement PAI-based treatment selection systems in that clinic, even in the absence of a randomized comparison between treatments, as the practical added value of the PAI will lie in its use and contribution to clinical practice. A PAI approach applied in a clinical setting will be inexpensive, such that even if the added value is not large, or is confined to patients whose PAI is large (in absolute value), the benefit to cost ratio will be favorable. But for now, this is an empirical question.

## Conclusions

The findings from this study suggest the promise of a simple method that combines prognostic and prescriptive information in a treatment selection approach that produces treatment recommendations for individual patients. Depressed patients who were randomized to the psychotherapy they were predicted to benefit the most from fared much better than those randomized to their non-optimal psychotherapy. When given a choice, most patients prefer psychotherapy compared to medications [35]. A data-informed choice between two equally effective psychotherapies not only improves outcomes for the individual patient, but also facilitates efficient use of mental health care services.

As inter-individual heterogeneity in treatment response tends to be large, and evidence-based predictive information is rarely used in decision-making, there exists a great opportunity for formal decision-making to improve patient outcomes in depression. Despite its promising nature and appeal, personalized medicine is a concept that is rarely applied in clinical practice. Developments in this direction have started to begin however, and a related, yet conceptually different approach to combining multiple moderators has recently been introduced by Kraemer [36]. In line with these developments, the present study encourages the introduction of treatment selection methods that can be used in regular mental health care, matching patients to the treatment option they are predicted to benefit the most from, increasing the *a priori* chance of recovery for the individual patient, and thereby advancing the goals of personalized medicine.
