# Correction: Predicting Optimal Outcomes in Cognitive Therapy or Interpersonal Psychotherapy for Depressed Individuals Using the Personalized Advantage Index Approach

**DOI:** 10.1371/journal.pone.0148835

**Published:** 2016-02-05

**Authors:** Marcus J. H. Huibers, Zachary D. Cohen, Lotte H. J. M. Lemmens, Arnoud Arntz, Frenk P. M. L. Peeters, Pim Cuijpers, Robert J. DeRubeis

There is an error in the first sentence of the third paragraph within the Results section. The correct sentence is: Female gender, being actively employed, low anxiety scores, the presence of a personality disorder and a high quality of life all predicted lower depression symptoms after treatment, irrespective of the therapy received.

There is an error in the second sentence of the second paragraph within the Discussion section. The correct sentence is: Female gender, active employment, low anxiety, the presence of a personality disorder and high quality of life were all indicators of a favorable prognosis.

There is an error in the last sentence of the second paragraph within the Discussion section. The correct sentence is: Carter et al. [10] found that patients with more comorbid personality disorder symptoms responded better to CT than to IPT, while we identified the presence of personality disorder only as a general prognostic factor.
